# The Role of Demoralization and Meaning in Life (DEMIL) in Influencing Suicidal Ideation Among Patients Affected by Chronic Pain: Protocol of a Single-Center, Observational, Case-Control Study

**DOI:** 10.2196/24882

**Published:** 2020-11-26

**Authors:** Alessandra Costanza, Vasileios Chytas, Viridiana Mazzola, Valérie Piguet, Jules Desmeules, Guido Bondolfi, Christine Cedraschi

**Affiliations:** 1 Department of Psychiatry Faculty of Medicine University of Geneva Geneva Switzerland; 2 Department of Psychiatry Santi Antonio e Biagio e Cesare Arrigo Hospital Alessandria Italy; 3 Division of Clinical Pharmacology and Toxicology Multidisciplinary Pain Center Geneva University Hospitals Geneva Switzerland; 4 Department of Psychiatry Service of Liaison Psychiatry and Crisis Intervention Geneva University Hospitals Geneva Switzerland

**Keywords:** suicide, suicide behavior, suicide attempt, suicidal ideation, chronic pain, demoralization, meaning in life, study protocol

## Abstract

**Background:**

Chronic pain is a significant risk factor for suicidal ideation (SI) and suicidal behavior (SB), including a 20%-40% prevalence rate of SI, a prevalence between 5% and 14% of suicide attempts, and a doubled risk of death by suicide in patients with chronic pain compared to controls. In most studies, associations between chronic pain and suicidality are robust, even after adjusting for the effect of sociodemographics and psychiatric comorbidity, and particularly for depressive conditions. A number of specific conditions that can modulate suicidality risk in patients with chronic pain have been investigated, but there is a need for their more specific characterization. Numerous recent studies have shown that demoralization and meaning in life (MiL) constructs affect suicidality as risk and protective factors, respectively. These constructs have been mainly investigated in patients with somatic illness and in community-dwelling individuals who may present with SI or SB independently of a psychiatric diagnosis of depression. However, a paucity of studies investigated them in suicidal patients affected by chronic pain.

**Objective:**

The primary objective of this project is to investigate the relationship between demoralization and MiL on SI risk in patients with chronic pain. The secondary objectives are (1) to test whether demoralization can occur independently of depression in patients with chronic pain and SI, (2) to examine whether the expected association between demoralization and SI may be explained by a sole dimension of demoralization: hopelessness, (3) to examine whether the presence of MiL, but not the search for MiL, is associated with less SI, and (4) to explore whether previously described MiL profiles (ie, high presence-high search, high presence-low search, moderate presence-moderate search, low presence-low search, and low presence-high search) emerge in our cohort.

**Methods:**

This project is a single-center, observational, case-control study—the Demoralization and Meaning in Life (DEMiL) study—conducted by the Division of Clinical Pharmacology and Toxicology, the Multidisciplinary Pain Centre, and the Service of Liaison Psychiatry and Crisis Intervention at the Geneva University Hospitals. Self- and hetero-administered questionnaires were conducted among patients and controls, matched by age and gender. The Ethics Committee of the Canton of Geneva approved the scientific utilization of collected data (project No. 2017-02138; decision dated January 25, 2018). Data have been analyzed with SPSS, version 23.0, software (IBM Corp).

**Results:**

From March 1, 2018, to November 30, 2019, 70 patients and 70 controls were enrolled. Statistical analyses are still in progress and are expected to be finalized in November 2020. To date, we did not observe any unfavorable event for which a causal relationship with the collection of health-related personal data could be ruled out. Results of this study are expected to form the basis for possible prevention and psychotherapeutic interventions oriented toward demoralization and MiL constructs for suicidal patients with chronic pain.

**Conclusions:**

The interest in exploring demoralization and MiL in chronic pain patients with SI arises from the common clinical observation that experiencing chronic pain often requires a revision of one’s life goals and expectations. Hence, the impact of chronic pain is not limited to patients’ biopsychosocial functioning, but it affects the existential domain as well. The major clinical implications in suicidal patients with chronic pain consist in trying to (1) delineate a more precise and individualized suicide risk profile, (2) improve detection and prevention strategies by investigating SI also in individuals who do not present with a clinically diagnosed depression, and (3) enhance the panel of interventions by broadening supportive or psychotherapeutic actions, taking into consideration the existential condition of a person who suffers and strives to deal with his or her suffering.

**International Registered Report Identifier (IRRID):**

DERR1-10.2196/24882

## Introduction

### Background

Chronic pain conditions are associated with an elevated risk of suicidal ideation (SI) and suicidal behavior (SB). Literature reviews indicate a 20%-40% prevalence rate of SI, a lifetime prevalence between 5% and 14% of suicide attempts, and a 2-fold higher risk of death by suicide in patients with chronic pain as compared to controls [1-7]. Most studies show that associations between chronic pain, SI, and SB are robust even after adjusting for the effect of sociodemographic factors and psychiatric comorbidity, particularly for depressive conditions [8-14]. A number of specific conditions that can modulate SI and SB risk in patients with chronic pain have been investigated, including pain characteristics, functional interference, illness beliefs, and access to opioids [2,3,15,16]. However, to improve SI and SB risk characterization in these patients, there is a need for the exploration of other potential risk and protection factors, in analogy to other chronic diseases [2,17]. To this end, the main goal of this project is to explore the role of two constructs, demoralization and meaning in life (MiL), in modulating SI among patients with chronic pain.

In an analogous study conducted in our institution, the same constructs [18-20], together with other psychological models [21-25], were investigated in a cohort of patients attending an emergency department. Demoralization and MiL concepts are theoretically and clinically connected, because meaninglessness is one of the five components of the definition of demoralization. Precisely, this link could be the key to utilize demoralization and MiL in supportive and psychotherapeutic interventions, that is, exploring with the patients the subconstructs of demoralization and the sense that the patient attributes to them, in order to restructure and reinforce an MiL that allows them to mitigate their suffering. The clinical interest in chronic pain patients with SI arises from the common clinical observation that experiencing chronic pain often requires a revision of one’s life goals and expectations [26]; hence, the impact of chronic pain is not limited to the patients’ biopsychosocial functioning but it affects the existential domain as well [16,27-30].

### The Demoralization Construct and Pain

The constitutive components of the demoralization construct, according Kissane and Clarke’s model, are loss of MiL, hopelessness or disheartenment, helplessness, sense of failure, and dysphoria [31,32] (see Costanza et al [18] for a detailed discussion on the demoralization construct). Various studies have indicated a possible relationship between demoralization and pain. For example, demoralization has been found to be associated with chronic facial pain [33], phantom tooth pain [34], and myofascial pain [35]. Greater pain intensity was found to be associated with the presence of demoralization in consultation-liaison psychiatry patients [36]. Another facet of painful illness, namely functional disability, was found to be correlated with demoralization in a sample of inpatients, independently of illness severity [37]. Loss of sense of dignity may also partially account for the association between physical problems and demoralization in advanced cancer patients [38]. Demoralization was found to be associated with lower pain reduction, reduced functional improvement, and decreased satisfaction among spine surgery patients [39].

### Demoralization and Suicidality

Two issues represent the impetus for investigating demoralization in individuals presenting with SB: (1) the relationship between demoralization and depression and (2) the role of one of the components of the demoralization definition (ie, hopelessness) in influencing SI and SB. By focusing on their phenomenological differentiation, most studies have suggested that demoralization is an independent condition distinguishable from depression [30,31,40]. In patients with cancer [41-45] and other medical diseases [46], there may be frequent overlaps between demoralization and depression, but demoralization can occur independently of depression and the two conditions are not necessarily linked by a hierarchical connection. By contrast, the relationship between demoralization and hopelessness is still being debated in the literature. Hopelessness is a dimension of the construct of demoralization rather than a synonym of demoralization [30,31,42,47-49]. However, for some authors it is still not clear whether definitions of demoralization containing hopelessness have predictive value over the construct of hopelessness alone [50]. This is particularly relevant for the evaluation of the role of demoralization in suicidality. Indeed, Beck first showed that hopelessness was an independent and more powerful predictor of suicidality than depression [51]. Therefore, more research is needed to better understand the relationship between demoralization and hopelessness, which has been found to be an independent mediator of SI and SB [31,52].

### The MiL Construct and Pain

A consensus in recent psychological research in defining MiL can be centered on two dimensions: coherence, or a sense of comprehensibility and one’s life making sense, and purpose, or a sense of core goals, aims, and direction in life. A third facet, significance, which focuses on values, worth, and importance of one’s life, is gaining increasing attention [53] (see Costanza et al [19,54] for an in-depth discussion on MiL). According to the largely utilized conceptualization of Steger, MiL is “the web of connections, understandings, and interpretations that help us comprehend our experience and formulate plans directing our energies to the achievement of our desired future” [55]. This theoretical model divides MiL into two subconstructs, specifically the *presence of* and *search for* MiL [53,56], which are not mutually exclusive [57]. Presence of MiL is rather uniformly thought to be beneficial [20,58]. In contrast, search for MiL appears more controversial, with some authors considering it the essence of human motivation [59], while others consider it a sign that one has lost meaning [60] or feels like one’s life has somewhat less meaning [58,61]. Different MiL profiles have been recently characterized in patients with chronic pain, resulting from the combination between low and high levels of presence of and search for MiL, which were associated with a unique adjustment outcome: patients having profiles with high scores of presence showed fewer depressive symptoms and greater life satisfaction [62]. Both MiL subconstructs have been found to be highly stable over time, suggesting that MiL may reflect a trait rather than a state aspect of individual functioning [63]. Among the psychotherapeutic interventions based on MiL, the *narrative* model, inspired by *logotherapy* described by Frankl [59], is the model for which clinical efficacy has been demonstrated both for patients with somatic conditions, such as palliative care patients [64], terminally ill cancer patients [65], and older adults with frailty [66], and also for individuals without an explicit somatic condition, such as older [67-69] and young adult patients [70], with or without depressive symptomatology.

### MiL and Suicidality

A number of studies have investigated MiL in individuals presenting SI and SB. A negative correlation between MiL—without distinction between presence of and search for MiL—and SI and SB has been found in undergraduate students [71-78], older adults [79-81], veterans [82], and patients affected by psychiatric disorders [77,81,83-85]. Overall, these studies suggested a protective role of MiL toward SI and SB.

To our knowledge, the constructs of presence of and search for MiL in individuals presenting SI and SB have only been explored in two studies that used the Meaning in Life Questionnaire (MLQ) [61], which was conducted in undergraduate students [86] and in soldiers returning from deployments [87]. In both, presence of MiL was correlated with a lower risk of SI and SB over time [86,87]. In contrast, the search for MiL subconstructs predicted a lower SI in one study [78] but a higher risk of suicide in another [87]. Notably, psychotherapeutic interventions targeting MiL were found to be effective in reducing suicide risk [88] and represent a promising therapeutic opportunity [82,85,87,89].

### Objectives

The primary objective of this project is to investigate the relationship between demoralization and MiL on SI risk in patients with chronic pain. The secondary objectives are (1) to verify if demoralization can occur independently of depression in patients with chronic pain and SI, (2) to examine if the expected association between demoralization and SI may be explained by a sole dimension of demoralization: hopelessness, (3) to examine whether the presence of MiL, but not the search for MiL, is associated with a lower SI, and (4) to explore if previously described MiL profiles [62,63] (ie, high presence-high search, high presence-low search, moderate presence-moderate search, low presence-low search, and low presence-high search) emerge in our cohort.

In summary, the demoralization and MiL constructs have been mainly investigated in patients with somatic illness and in community-dwelling individuals who may present with SI or SB independently of a psychiatric diagnosis of depression. Only a very small number of studies investigated them in patients affected by chronic pain presenting with SI. This population can provide the opportunity to analyze relationships between demoralization, depression, and hopelessness as well the role of the presence of MiL and search for MiL subconstructs in influencing SI and SB [26].

## Methods

### Participant Confidentiality and Data Handling

Participants’ dignity, privacy, and health were preserved and respected, guaranteeing participants full anonymity if study data are to be presented at scientific meetings or published in scientific journals. Individual participant medical information remains confidential and will not be disclosed to third parties. Participant data were anonymized with coded identification numbers that correspond to the participants’ medical records using a progressive numbering system (ie, 001, 002, 003, etc). All participant-related documents were stored in a secure and locked location. Electronic data were protected using strong password encryption. Data generation, transmission, storage, and analysis of health-related personal data followed the current Swiss legal requirements for data protection and were performed according to Human Research Ordinance Article 5.

### General Project Design and Procedures

This project is an observational study—the Demoralization and Meaning in Life (DEMiL) study—conducted by the Division of Clinical Pharmacology and Toxicology and the Service of Liaison Psychiatry and Crisis Intervention at the Multidisciplinary Pain Centre (MPC) of the Geneva University Hospitals (see Tables 1 and 2 [45,61,90-93]).

**Table 1:** Methodological tools to assess primary outcomes.

Variable and instrument		Main characteristics of the instrument
**Suicidal ideation (SI)**		
	Item No. 9 of the Beck Depression Inventory-II [91], French-validated version	▪Self-report multiple-choice inventory ▪Indicator of the severity of depression ▪Standard cutoff scores: 0-13 (minimal depression), 14-19 (mild depression), 20-28 (moderate depression), and 29-63 (severe depression) ▪21 items, each one rated on a 4-point scale, ranging from 0 to 3, based on severity of the item ▪About 5-10 minutes to complete
	Scale for Suicidal Ideation [92], French-validated version	▪Scale based on a semistructured interview with the patient ▪Indicator of characteristics and severity of an individual's plans and wishes to commit suicide ▪19 items, each one rated on a 3-point scale, ranging from 0 to 3 (except for item 13, which is rated on a 4-point scale), based on severity of the item ▪Total score for the 19 items: minimum score 0 and maximum score 38 (higher scores indicate greater SI) ▪About 5-10 minutes to complete
**Demoralization**		
	Demoralization Scale [45]	▪Self-reported multiple-choice inventory ▪Indicator of the presence and severity of demoralization; standard cutoff score of >30 indicative of severe demoralization ▪24 items, each with a 5-point response scale describing the frequency of occurrence for each item: 0 (never), 1 (seldom), 2 (sometimes), 3 (often), and 4 (all the time) ▪5-factor structure (subscales): loss of meaning and purpose (5 items), dysphoria (5 items), disheartenment (6 items), helplessness (4 items), and sense of failure (4 items) ▪About 5-10 minutes to complete
**Meaning in life**		
	Meaning in Life Questionnaire (MLQ) [61], French-validated version	▪Self-reported multiple-choice inventory ▪Measure of the *presence of* (5 items) and the *search for* (5 items) meaning in life; the MLQ does not have cutoff scores, because it is intended to measure meaning in life across the complete range of human functioning ▪10 items, each rated on a 7-point response scale, from *absolutely true* to *absolutely untrue* ▪About 3-5 minutes to complete

**Table 2:** Schedule of assessments (flow of research project).

Assessments		Occurrence of assessment^a^ at each project period		
		Before initial routine visit	Initial routine visit: around 15 days after having received MPC^b^ routine screening self-administered questionnaires	Second visit: 1-7 days after initial MPC routine visit
**MPC routine screening and postscreening self-administered questionnaires (completed at home)**				
	Demographic data: gender, age, marital status, language, education, professional activity, and insurance claims	x		
	Pain data: localization (pain drawings), intensity (Visual Analog Scale), duration, characteristics (McGill Pain Questionnaire), and aggravating and alleviating factors	x		
	Severity disability (*Oswestry Disability Index*)	x		
	Quality of life (36-Item Short Form Survey) [93]	x		
	Patient expectations about MPC	x		
	Illness beliefs (Brief Illness Perception Questionnaire–Revised)	x		
	Demoralization (Demoralization Scale) [45]	x		
	Severity of depression (Beck Depression Inventory-II [BDI-II]) [91]	x		
	Suicidal ideation (item No. 9 of the BDI-II) [91]	x		
MPC routine visit + study information to patient (written communication)			x	
**Study visit (required time 1 hour)**				
	Informed consent and inclusion			x
	Clinical evaluation of suicidal ideation^c^			x
	Characteristics and severity of suicidal ideation (Scale for Suicidal Ideation)^c^ [92]			x
	Meaning in life (Meaning in Life Questionnaire) [61]			x
	Clinical diagnostic and exclusion of major depressive disorder and/or other psychiatric comorbidities (clinical interview and structured interview by French 5.0.0 version of the Mini-International Neuropsychiatric Interview [90])			x

^a^x indicates that the assessment was performed, while a blank cell indicates that it was not.

^b^MPC: Multidisciplinary Pain Centre.

^c^This assessment was only performed in patients with suicidal ideation: those with a score between 1 and 3 in question 9 of the BDI-II.

The research project was carried out in accordance with the research plan and Swiss legal and regulatory requirements, in agreement with the principles stated in the current version of the Declaration of Helsinki, the Essentials of Good Clinical Practice, issued by Public Health Switzerland. Self-administered questionnaires were sent out to each participant before their first routine MPC visit. The utilization for scientific purposes of data collected with these questionnaires was approved by the Ethics Committee (EC) of the Canton of Geneva (project No. 2017-02138, decision dated January 25, 2018). During their routine MPC visit, about 15 days after receiving the questionnaires, all participants received a written communication informing them about the research project and were able to pose questions or raise concerns with the project sponsor. Each participant was given at least 24 hours to review the written communication before being asked to sign the informed consent document, which took place during a subsequent study visit. A qualified team member reviewed the questionnaires for presence of SI and communicated relevant information about this study to the participants. Appropriate measures were taken for cases where severe SI was identified, including, if necessary, accompanying the patient to the psychiatric emergency ward. About 1-7 days after the routine MPC visit, patients who had agreed to be included in the study had a second study visit where they signed the informed consent form. All patients underwent completion of the MLQ [61].

The desire and possibility of being included in the study did not influence in any way the patients’ overall evaluation or treatment. All patients received the appropriate treatment for their clinical situation as recommended by the MPC, which included pharmacological modifications, individual or group psychiatric treatment, and/or physical treatment.

They also underwent a clinical interview and a structured diagnostic interview to screen for psychiatric diagnoses, including major depressive disorder, according to the DSM-IV (Diagnostic and Statistical Manual of Mental Disorders, fourth edition) and the French version 5.0.0 of the Mini-International Neuropsychiatric Interview (MINI) [90]. Patients with SI—as identified by their positive response to question 9 in the Beck Depression Inventory-II (BDI-II) [91]—whose scores ranged from 1 to 3, additionally underwent a clinical evaluation of SI by a qualified team member using the Scale for Suicidal Ideation (SSI) to assess characteristics and severity of SI [92]. The study visit took approximately 1 hour to complete.

### Recruitment and Screening

Participants were enlisted by ongoing recruitment through a project anchored in daily clinical practice at the MPC. To control for potential confounding individual differences, we screened for quality of life, assessed using the 36-Item Short Form Survey [93]; illness beliefs, assessed using the Brief Illness Perception Questionnaire–Revised [94]; pain characteristics; and drug therapy.

Inclusion criteria consisted of patients affected by chronic pain referred to the MPC and presenting with SI and control subjects not presenting with SI; groups were matched for age and gender. As the MPC is a third-line ambulatory referral center, most of the patients referred to the center by their treating physicians suffer from chronic neuropathic or nociceptive pain. Over the course of a typical year, about 40%-50% of incoming patients suffer from neuropathic pain; about 40% have osteoarticular pain, mostly lower back pain, fibromyalgia, and joint pain; while the remaining 10%-20% suffer from chronic visceral pain or from single-fraction radiotherapy cancer-related pain. Participants had to provide informed written consent and be 18 years of age or older. Exclusion criteria included patients with an insufficient comprehension of the French language and those affected by dementia, psychotic disorder, or borderline personality disorder. In cases of withdrawal of informed consent or noncompliance, participants were excluded from the study. If participants withdrew due to personal reasons, they were asked to inform the project leader about their withdrawal. Additional participants were recruited to replace those who had withdrawn.

### Assessment of Primary and Secondary Outcomes

Regarding primary endpoints, we expected to observe a higher score on the Demoralization Scale (DS) [45] and a lower score on the MLQ [68] for patients with SI (presence and severity) as measured by question 9 of the BDI-II [91] and the SSI [92]. The DS and BDI-II were administered as part of routine questionnaires. In order to assess our primary endpoints, we utilized the instruments outlined in Table 1.

Regarding secondary endpoints, we expected that patients with SI (presence and severity) would have (1) a higher DS score but not necessarily a higher BDI-II score, (2) a higher score for search for MiL and a lower score for presence of MiL, and (3) an MiL profile of combined presence and search, with higher scores for search for MiL and lower scores for presence of MiL.

The following tools were used to assess secondary endpoints:

1. The BDI-II was utilized to assess depression severity [91]. The presence or absence of depression was determined during a clinical interview using a structured diagnostic interview to screen psychiatric diagnoses, according the French version 5.0.0 of the MINI [90] for DSM-IV disorders.
2. The MLQ [68] was used to define MiL profiles and categorize them as high presence-high search, high presence-low search, moderate presence-moderate search, low presence-low search, or low presence-high search.
3. The sense of hopelessness was determined with the
  helplessness
  subscale of the DS [45], which comprises four items to which responses are given using a 5-point Likert scale; subscale scores range from 0 to16, and high scores indicate a strong sense of helplessness. It has been theorized that helplessness is a subdivision of hopelessness [21] and, from a dimensional perspective, leaving helplessness untreated may lead to hopelessness, which is in turn strongly associated with suicidal intent [31,32].

### Definition, Assessment, and Reporting of Serious Events

A serious event (SE) is defined as any unfavorable event for which a causal relationship with the collection of health-related personal data cannot be ruled out and which (1) requires hospitalization or prolongation of hospitalization, (2) results in persistent or significant disability or incapacity, or (3) is life-threatening or results in death. When an SE occurs, the research project is put on hold and the EC is informed.

This would be followed by an assessment by the project leader with regard to the project-specific measure relationship according to the following definitions:

1. Unrelated: the occurrence of the event has no temporal relationship to the project-specific measures applied and can be explained by the underlying disease or other factors.
2. Related: there is a plausible temporal relationship between the occurrence of the event and the project-specific, applied measures and cannot be explained by the underlying disease or other factors.

SEs are documented in each participant’s case report form and source document and reported to the EC within 7 days. A report would be submitted to evaluate the relationship between our study and the event, explaining how the event affected the project and, if applicable, outline further protective measures to prevent reoccurrence.

### Statistical Methodology

Assuming a 5% margin of error; a confidence level of 95%; a target population size of 400, as around 400 new patients are evaluated annually at the MPC; and a response distribution of 50%, then the minimum sample size required for this study is approximately 200: 100 presenting SI and 100 nonpresenting SI participants matched by age and gender. Questionnaire data were manually entered into an SPSS, version 23.0, database (IBM Corp) [95] using double data entry to minimize errors. Each participant was assigned a unique code that was recorded on their questionnaire. Questionnaires and demographic data were analyzed with SPSS, version 23.0, software (IBM Corp) [95].

Descriptive statistical analyses were conducted to compute means and SDs for numerical variables and frequencies (%) for categorical variables. The chi-square test or Fisher exact test was used for categorical variables and checked for matching between groups regarding age, gender, and years of education. To test our main hypothesis, repeated-measures analyses of variance were conducted to test for significant interaction between DS and MLQ variables and group factors (SI vs non-SI). The post hoc Tukey multiple-comparison test was used to find significant differences between means. In addition, hierarchical multiple regressions were used to test for predictive effects of MLQ and DS scores. Multivariate linear and logistic regression models were employed to explore the associations between MLQ score, DS score, and suicidal risk. Covariates were introduced into our model using a stepwise backward procedure to attain a parsimonious model to test effect modifications related to sociodemographic (ie, gender, education, drug therapy, etc) and pain characteristics. All results are reported with a significance threshold of 2-sided *P* values of less than .05 and effect size. Missing value analysis was performed for all questionnaire scores to check for significant patterns. Where no patterns could be identified, the random missing values were replaced by the mean.

## Results

From March 1, 2018, to November 30, 2019, 70 patients and 70 control participants were enrolled. Statistical analyses are still in progress and are expected to be finalized in December 2020. We did not observe any unfavorable event for which a causal relationship with the collection of health-related personal data could be ruled out. The results of this study will be used to develop new proposals for prevention and psychotherapeutic interventions oriented toward demoralization and MiL in patients with chronic pain, which will be based on the model of already-existing psychotherapeutic interventions for patients with other somatic conditions.

## Discussion

The interest in exploring demoralization and MiL in chronic pain patients with SI arises from the common clinical observation that experiencing chronic pain often requires a revision of one’s life goals and expectations. Different coping mechanisms have been proposed for chronic pain [96,97]. However, the impact of chronic pain is not limited to patients’ biopsychosocial functioning, but it affects the existential domain as well. The major clinical implications in suicidal patients with chronic pain consist in trying to (1) delineate a more precise and individualized suicide risk profile, (2) improve detection and prevention strategies by investigating SI also in individuals who do not present with a clinically diagnosed depression, and (3) enhance the panel of interventions by broadening supportive or psychotherapeutic actions. The two constructs of MiL and demoralization are intimately and opposingly linked because meaninglessness is one of the subconstructs underlying the construct of demoralization [31,32]. In the specific context of chronic pain, this link precisely could be key to utilize these theoretical models in psychotherapeutic interventions based on the *narrative* model [58,59,66-70], that is, to explore with patients the subconstructs of demoralization and the sense that they attribute to them in order to restructure and reinforce an MiL that allows them to mitigate their suffering, taking into consideration the existential condition of a person who suffers and strives to deal with their suffering.

## Abbreviations

BDI-II: Beck Depression Inventory-II
DEMiL: Demoralization and Meaning in Life
DS: Demoralization Scale
DSM-IV: Diagnostic and Statistical Manual of Mental Disorders, fourth edition
EC: Ethics Committee
MiL: meaning in life
MINI: Mini-International Neuropsychiatric Interview
MLQ: Meaning in Life Questionnaire
MPC: Multidisciplinary Pain Centre
SB: suicidal behavior
SE: serious event
SI: suicidal ideation
SSI: Scale for Suicidal Ideation
